# Specific guidelines for assessing and improving the methodological quality of economic evaluations of newborn screening

**DOI:** 10.1186/1472-6963-12-300

**Published:** 2012-09-04

**Authors:** Astrid Langer, Rolf Holle, Jürgen John

**Affiliations:** 1 Institute of Health Economics and Health Care Management, Munich School of Management, Ludwig-Maximilians Universität München, Munich, Germany; 2 Institute of Health Economics and Health Care Management, Helmholtz Zentrum München – German Research Centre for Environmental Health, Neuherberg, Germany

## Abstract

**Background:**

Economic evaluation of newborn screening poses specific methodological challenges. Amongst others, these challenges refer to the use of quality adjusted life years (QALYs) in newborns, and which costs and outcomes need to be considered in a full evaluation of newborn screening programmes. Because of the increasing scale and scope of such programmes, a better understanding of the methods of high-quality economic evaluations may be crucial for both producers/authors and consumers/reviewers of newborn screening-related economic evaluations. The aim of this study was therefore to develop specific guidelines designed to assess and improve the methodological quality of economic evaluations in newborn screening.

**Methods:**

To develop the guidelines, existing guidelines for assessing the quality of economic evaluations were identified through a literature search, and were reviewed and consolidated using a deductive iterative approach. In a subsequent test phase, these guidelines were applied to various economic evaluations which acted as case studies.

**Results:**

The guidelines for assessing and improving the methodological quality of economic evaluations in newborn screening are organized into 11 categories: “bibliographic details”, “study question and design”, “modelling”, “health outcomes”, “costs”, “discounting”, “presentation of results”, “sensitivity analyses”, “discussion”, “conclusions”, and “commentary”.

**Conclusions:**

The application of the guidelines highlights important issues regarding newborn screening-related economic evaluations, and underscores the need for such issues to be afforded greater consideration in future economic evaluations. The variety in methodological quality detected by this study reveals the need for specific guidelines on the appropriate methods for conducting sound economic evaluations in newborn screening.

## Background

Where resources needed for the production of health benefits are scarce, decision makers need to consider both the effectiveness and the cost-effectiveness of health interventions. Economic evaluation is the comparative analysis of alternative interventions with regard to both their costs and outcomes [1]. Although economic evaluations are used to inform decision making in health care, several studies have shown that economic evaluations differ markedly in quality [2-4]. Common methodological flaws in economic evaluations relate to study design, data collection and analysis, and interpretation or reporting of results [5]. In order to assess the methodological rigour of economic evaluations, various instruments have been developed. To date, two of these instruments have received more scrutiny than others [6], namely the “Guidelines for authors and peer reviewers of economic submissions to the BMJ“ [7] and the “Consensus on Health Economic Criteria (CHEC) list for assessment of methodological quality of economic evaluations“ [8]. Whereas the latter is a specific list of criteria for conducting systematic reviews of trial-based economic evaluations, the former is a general checklist for assessing economic evaluations. However, both the study questions and the inclusion criteria generally require additional disease-specific guidelines in order to assess and improve the methodological quality of economic evaluations in a specific disease area [9]. For instance, in order to support effective decision making, the Canadian Agency for Drugs and Technologies in Health (CADTH) published oncology guidelines which provide specific guidance for conducting high-quality economic evaluations of oncology products [10].

The uniqueness of child health and associated methodological challenges in conducting economic evaluations have already been pointed out [11]. Specific methodological challenges also emerge in economic evaluations of newborn screening. For instance, there is a lack of adequate and robust long-term data from which useful evidence can be produced [12,13]. Besides the dearth of long-term follow-up studies of newborn screening programmes and the associated extrapolation of short-term data to capture a lifetime time horizon, other challenges pertain to the problem of appropriate discounting, the use of quality adjusted life years (QALYs) in newborns, and which costs and outcomes need to be considered in a full evaluation of newborn screening programmes. Because of the increasing scale and scope of such programmes, a better understanding of the methods of high-quality economic evaluations may be crucial for both producers/authors and consumers/reviewers of newborn screening-related economic evaluations in order to assure high quality allocation decisions in newborn screening. The aim of this study was therefore to establish specific guidelines for assessing and improving the methodological quality of economic evaluations in newborn screening. These guidelines are designed for both producers/authors and consumers/reviewers of economic evaluations of newborn screening, and are suitable for trial-based and model-based economic evaluations. Additionally, the guidelines were tested with respect to various economic evaluations in the areas of newborn hearing screening and newborn screening for medium-chain acyl–CoA dehydrogenase deficiency (MCADD) using tandem mass spectrometry (MS/MS).

## Methods

In order to identify and review existing guidelines for assessing the quality of economic evaluations, several databases and the internet were searched. Using a deductive iterative process, data from textbooks on health economic evaluation [1,14,15], principles of good practice published by different ISPOR Task Forces [16,17], and from several guidelines, checklists and criteria lists [7,8,18-22] were extracted qualitatively based on general categories identified in previous work [19]. After data extraction, the relevance of each category and item within it was discussed in relation to the development of specific guidelines to assess and improve the methodological quality of economic evaluations in newborn screening. The final set of guidelines was developed based on majority consensus.

In order to test the specific guidelines, they were applied to economic evaluations which were identified through a systematic review of the literature: economic evaluations of newborn hearing screening and economic evaluations of newborn screening for inherited metabolic disorders including MCADD by MS/MS. The following databases were searched from inception to April 2012 for relevant studies: HTA database, NHS Economic Evaluation Database, Pediatric Economic Database, PubMed, Web of Science. The literature search for economic evaluations of newborn hearing screening used the following terms: (“newborn” or “neonatal” or “infant”) combined with (“economic” or “cost”) and (“hearing” or “deaf”) and “screening”. The literature search for economic evaluations of newborn screening for inherited metabolic disorders including MCADD by MS/MS used the following terms: (“newborn” or “neonatal” or “infant”) combined with (“economic” or “cost”) and (“screening” or “tandem mass spectrometry” or “MS/MS screening”) and (“metabolic disorders” or “MCADD” or “MCAD” or “medium-chain acyl-CoA dehydrogenase deficiency”). The searches were limited by study design (cost analysis, cost-minimization analysis, cost-effectiveness analysis, cost-utility analysis, and cost-benefit analysis), year (2000 – 2012) and language of publication (English, French or German). In addition, the economic analyses should be fully described and transparent. Further studies were identified by examining the reference lists of several systematic reviews and health technology assessments in the areas of newborn hearing screening [23-25] and newborn screening for inherited metabolic disorders including MCADD by MS/MS [12,13,26,27]. The full list of sources and the search strategy are available from the authors.

## Results

The guidelines for assessing and improving the methodological quality of economic evaluations in newborn screening are organized into 11 categories: “bibliographic details”, “study question and design”, “modelling”, “health outcomes”, “costs”, “discounting”, “presentation of results”, “sensitivity analyses”, “discussion”, “conclusions”, and “commentary”. The full set of guidelines is presented in Table 1.

**Table 1:** Specific guidelines for assessing and improving the methodological quality of economic evaluations of newborn screening

**A. Bibliographic details**	
1.	Author(s)
2.	Institutional affiliation of author(s)
3.	Source of funding
4.	Title
5.	Source of publication
6.	Publication type
**B. Study question and design**	
1.	Study question
2.	Intervention
3.	Control
4.	Target population
5.	Time horizon
6.	Setting
7.	Perspective
8.	Study design
9.	Type of economic evaluation
10.	Study population
11.	Primary outcome measure
**C. Modelling**	
1.	Model type
2.	Model structure
3.	Model assumptions
4.	Sources used to develop and/or populate the model
5.	Cycle length
6.	Health states and transition
7.	Model validation
**D. Health outcomes**	
1.	Health outcomes measured in natural units
	*a. Type of health outcome measure used in the economic evaluation*
	*b. Method of measurement*
	*c. Source of data*
2.	Health outcomes adjusted by utility weights or health state preference scores
	*a. Type of health outcome measure used in the economic evaluation*
	*b. Method of valuation*
	*c. Source of data*
3.	Health outcomes measured in monetary units
	*a. Type of health outcome measure used in the economic evaluation*
	*b. Method of valuation*
	*c. Source of data*
4.	Intermediate health outcomes
	*a. Type of health outcome measure used in the economic evaluation*
	*b. Method of measurement*
	*c. Source of data*
5.	Non-health outcomes
	*a. Type of health outcome measure used in the economic evaluation*
	*b. Method of measurement*
	*c. Source of data*
**E. Costs**	
1.	Patient-related costs
	*a. Cost categories considered in the economic evaluation*
	*b. Measurement of resource data*
	*c. Valuation of resource data*
	*d. Source of data*
2.	Programme-related costs
	*a. Cost categories considered in the economic evaluation*
	*b. Measurement of resource data*
	*c. Valuation of resource data*
	*d. Source of data*
**F. Discounting**	
1.	Discount rate for costs
2.	Discount rate for health outcomes
3.	Justification of discount rates
**G. Presentation of results**	
1.	Absolute and incremental health outcomes per newborn
2.	Absolute and incremental costs per newborn
3.	ICER for the primary outcome measure
4.	Present values and trends of costs and health outcomes at the population level
5.	Present values and trends of costs at the population level differentiated by payer
6.	Coverage of screening
**H. Sensitivity analyses**	
1.	Parameter uncertainty
2.	Modelling uncertainty
3.	Methods of sensitivity analyses
4.	Results of sensitivity analyses
**I. Discussion**	
1.	Limitations of the study
2.	Generalizability and transferability of the economic evaluation results
3.	Ethical and distributional issues
**J. Conclusions**	
1.	Validity of conclusions with regard to the results of the economic evaluation
2.	Validity of conclusions with regard to the objective of the economic evaluation
**K. Commentary**	
1.	Selection of comparators
2.	Validity of estimate of measure of effectiveness
3.	Validity of estimate of measure of health outcome
4.	Validity of estimate of costs
5.	Other issues
6.	Implications of the economic evaluation

### A. Bibliographic details

In addition to the common bibliographic details (author(s), title, and source of publication), details on the publication type (e.g., peer-reviewed journal article, non-peer-reviewed journal article, abstract, technical report, book, or book section) should be provided in order to obtain information on the methods of quality control during the publication process. Furthermore, the institutional affiliation of author(s) and the source of funding should be stated because both items can act as an indicator for potential conflicts of interest.

### B. Study question and design

First, a clearly defined and answerable study question should be posed. This should encompass a detailed description of the screening intervention under evaluation and its comparator. Furthermore, the target population of the screening intervention and the time horizon over which costs and health outcomes are likely to accumulate should be stated. The perspective and the time horizon of the analysis should be clearly stated, and the reasons underlying these choices should be made transparent. Fixing these boundaries of the study determines which elements of costs and benefits should be included in the economic evaluation. If the primary purpose of the economic evaluation is to assist health policy makers in making optimal societal decisions, a societal perspective should be chosen, i.e. all relevant short- (costs of screening and diagnostic follow-up) and long-term costs (costs for therapy, rehabilitation, education and social services, and lost productivity), and consequences such as morbidity or mortality, should be included. In the case of newborn screening programmes, this societal perspective usually implies that a lifetime time horizon should be adopted. To judge the generalizability and transferability of the results of economic evaluation, the setting, including time and place of intervention, should be described in detail, because the type of setting (e.g., inpatient or outpatient) can have a great influence on screening coverage. Finally, the study design (clinical study or decision-analytic model), the type of economic evaluation (cost-consequences analysis, cost-minimization analysis, cost-effectiveness analysis, cost-utility analysis, and cost-benefit analysis), and the primary outcome measure used in the economic evaluation should be stated, and the study population should be described in detail to assess the generalizability of economic evaluation results with regard to the target population of the screening intervention.

### C. Modelling

Economic evaluations based on decision-analytic models synthesize the available evidence systematically and provide projected estimates of long-term outcomes when long-term data are missing. Thus, decision-analytic models can support policy decisions for newborn screening programmes [28]. First, the model type (cohort model or patient-level simulation model) should be specified. Furthermore, the model structure should be described in detail, including statement of decision problem and scope, and it should be appropriate for addressing the study question. In addition, all model assumptions and associated choices should be stated and justified, and all sources used to develop and/or populate the model should be stated and justified. Furthermore, in Markov models, information on cycle length, and health states and transitions should be provided. Finally, with regard to model validation, internal and external validations should be described. A more detailed framework for assessing the quality of decision-analytic models is available [21], which is based on a comprehensive review of the respective literature [29]. It is recommended that producers/authors and consumers/reviewers of newborn screening-related economic evaluations consult these or comparable good modelling practice guidelines for a more detailed assessment of the transparency and quality of model-based economic evaluations.

### D. Health outcomes

Initially, it is necessary to determine which type of health outcome measure was used in the economic evaluation to assess whether all relevant consequences of the condition under evaluation were considered. The choice of health outcome measure should be based on the condition(s), intervention, and health policy setting and audience. Common types of health outcome measure used in newborn screening-related economic evaluations are health outcomes measured in natural units (e.g., life-years gained or cases correctly diagnosed), and preference-based health outcomes such as the quality adjusted life year (QALY). In general, health outcome measures should be used that are relevant to the patient. Especially with regard to economic evaluations of newborn screening for inherited metabolic disorders, appropriate preference-based health outcomes which integrate mortality and morbidity in a single outcome measure should be used to value meaningful differences between MS/MS and alternatives in terms of health-related quality of life. Furthermore, in contrast to measuring outcome by the number of cases correctly diagnosed, preference-based health outcomes such as QALYs can be used to compare screening interventions that are targeted at different disorders. However, the use of QALYs in child health care poses serious methodological challenges which must be considered carefully in the search for an appropriate outcome measure [30,31]. Other health outcomes in newborn screening-related economic evaluations are intermediate health outcomes, non-health outcomes, or health outcomes measured in monetary units. In all cases, the type of health outcome or effectiveness measure influences its measurement and valuation. The methods used to measure and value health outcomes should be the most appropriate ones for the condition under evaluation and the study question, and should be stated for reasons of transparency and comparability of economic evaluations. Finally, the source of data used to calculate the health outcomes should be provided as well, including the evidence of effectiveness. One of the main weaknesses in economic evaluations of newborn screening is usually in the area of epidemiology [12]. There is a lack of data to directly compare health outcomes for both screened and unscreened cohorts. Furthermore, readers should be aware that some sources of data such as systematic reviews might be more valid than others such as expert opinions. To validate data and modelling assumptions, epidemiologists and clinicians should be consulted.

### E. Costs

Contrary to many other health technologies, interventions in newborn screening generate costs at the administrative level and at the level of the individual patient. Patient-related costs consist of direct medical costs (screening costs, treatment costs, and downstream health costs, such as monitoring and managing of cases detected), direct non-medical costs (e.g., travel expenses and time costs of parents accompanying their child, or costs associated with institutional care and special education), and indirect costs (costs for lost productivity). With regard to indirect costs in economic evaluations of newborn screening, it is essential to value both the patient and the caregiver time. A critical review of existing valuation methods can be found elsewhere [32]. Programme-related costs are all “costs incurred at the administrative levels outside the point of delivery of health care to beneficiaries“ [33], such as costs for the implementation, organization, administration, monitoring, and evaluation of the screening programme and costs for tracking. In general, all resources that are relevant to the study perspective should be identified, measured, and valued. For instance, additional health care costs prior to diagnosis may be included for conditions not screened for [13], or unrelated costs that accrue from the intervention during life-years gained may be considered as well, an issue which is controversial in the literature [34-39]. Both the costing and the valuation method (e.g., adjustment for inflation) used should be stated and justified, and the sources used to obtain data on resource utilisation and unit costs should be reported. In order to enhance generalizability and transferability, quantities and unit costs should be reported separately.

### F. Discounting

In newborn screening-related economic evaluations with a longer time horizon than one year, costs and health outcomes should be discounted. However, discount rates can have a great influence on the results of an economic evaluation [40,41]. For instance, studies which discount costs more than they discount health outcomes will result in a relatively low ICER [13]. Therefore, the choice of discount rates should be stated and justified: both a discount rate for costs and a discount rate for health outcomes should be provided. Furthermore, a justification of the discount rates should be given – i.e., the rationale for the choice of discount model and discount rates in the base case and in the sensitivity analyses, and for the use of different or common discount rates for costs and health outcomes should be provided.

### G. Presentation of results

First, both the costs and the health outcomes should be presented per newborn, and in both disaggregated and aggregated forms. Furthermore, an incremental cost-effectiveness ratio (ICER) should be provided for the primary outcome measure used in the economic analysis. Present values and trends of costs and health outcomes at the population level should be reported as well. Additionally, in terms of costs, present values and trends should be differentiated by payer because in newborn screening costs are borne by different payers (e.g., health insurance, patient, or state and federal government). Finally, the coverage of screening should be reported because it is one of the key parameters driving the cost-effectiveness of alternative screening strategies.

### H. Sensitivity analyses

In the area of newborn screening for inherited metabolic disorders, various factors, such as prevalence of the disorder, or frequency of adverse outcomes, influence the ICER [42], and therefore should be varied in sensitivity analyses in order to assess the impact on economic evaluation results and conclusions. Parameter uncertainty should be addressed, including a statement and justification of the ranges or distributions of parameters used for sensitivity analyses. In model-based economic evaluations, modelling uncertainty should be investigated as well because structure, methods, and assumptions of models are subject to uncertainty. Furthermore, methods (univariate or multivariate sensitivity analyses, probabilistic sensitivity analyses, threshold analyses and analyses of extremes or best/worst case analyses) and results of sensitivity analyses should be reported.

### I. Discussion

First, the limitations of the study should be discussed. For instance, in newborn screening-related economic evaluations, one of the main constraints and weaknesses refers to the lack of long-term data [12]. Second, both generalizability and transferability of the study results should be discussed, which relate to the extent to which the economic evaluation results are applicable to different settings. Methodological (e.g., perspective, discount rate), health care system (e.g., absolute and relative prices in health care), and population characteristics (e.g., disease prevalence) are factors that influence the transferability of economic evaluation results [43] in general as well as in newborn screening. Finally, ethical and distributional issues should also be considered, because both are essential for economic evaluations with respect to rare and disabling conditions. In the case of economic evaluations of newborn screening, ethical issues which might be of concern to users include the principles of beneficence (“finding the evidence of benefit”), non-maleficence (“finding the harm”), autonomy (“the right to choose, and the protection of those with diminished autonomy”), and justice (“distributive justice”) [44]. For instance, ethical challenges emerging from the use of QALYs for disabling conditions should be addressed [45]. Other ethical issues are associated with the use of MS/MS: detection of diseases for which there is no (effective) treatment, detection of diseases whose natural history is not (well) understood, detection of maternal variance, detection of carriers, and finding the evidence of benefit [44]. However, benefits from the early and correct diagnosis of a newborn with a largely untreatable disorder relate to the prevention of misdiagnosis, reassurance of parents, and enabling future reproductive choices [44]. In economic evaluations of newborn screening for inherited metabolic disorders, ethical issues regarding antenatal screening may also be included [13]. Furthermore, the distribution of costs and benefits should be considered [46].

### J. Conclusion

The conclusions should follow from the results of the economic evaluation and should answer the study question.

### K. Commentary

The commentary of the consumer/reviewer of the economic evaluation should be a critical summary of the overall reliability and generalizability of the economic evaluation and include the following issues: Selection of comparators, validity of estimate of measure of effectiveness, validity of estimate of measure of health outcome, validity of estimate of costs, other issues, and implications of the economic evaluation. These issues were adapted from the NHS EED structured abstract format [47].

### Application of the guidelines for assessing and improving economic evaluations in newborn screening

In the following, the experiences of applying the guidelines to economic evaluations in the areas of newborn screening for inherited metabolic disorders including MCADD by MS/MS and newborn hearing screening are reported.

### Economic evaluations of newborn hearing screening

In the area of newborn hearing screening, the guidelines were applied to 21 economic evaluations [23-25,48-65], of which an overview is provided in Additional file 1. These studies were heterogeneous with regard to target population (all newborns or newborns with risk factors), screening technology (otoacoustic emission (OAE) or automated auditory brainstem response (AABR)), screening strategy (universal screening or risk screening), number of stages in the initial screening process (one or two), detection of hearing loss (unilateral or bilateral), setting (inpatient or outpatient), cost categories considered (development/implementation/organization/monitoring of the screening programme, tracking, screening tests, diagnostic follow-up, treatment for detected cases of hearing disorder, patient transportation for diagnostic procedures and treatment, education, work time loss for parents/adults with hearing disorders, productivity loss due to hearing disorders/premature mortality), and comparators. Most of the studies evaluated different screening technologies [25,48-51,54-59]. Five economic evaluations compared different screening strategies [23,24,60-62]. Two studies analyzed different screening devices [53,64]. Two other studies examined different settings [52,65], and one study compared subjective and objective screening tests [63]. Six of the economic evaluations were from the United States [51,55-57,60,62], six from Germany [23,48,53,61,64,65], three from the United Kingdom [52,54,63], two from Taiwan [58,59] and Canada [25,50], and one each from Australia [24] and the Netherlands [49].

The results of the application of the guidelines revealed that most of the economic evaluations met the following items:

· Study question (B.1.)

· Intervention (B.2.)

· Control (B.3.)

In contrast, the following items were inadequately met or not considered at all:

· Model validation (C.7.)

· Health outcomes measured in natural units (D.1.), adjusted by utility weights or health state preference scores (D.2.), or measured in monetary units (D.3.)

· Measurement (E.1.b. and E.2.b.) and valuation (E.1.c. and E.2.c.) of resource data

· Justification of discount rates (F.3.)

· ICER for the primary outcome measure (G.3.)

· Modelling uncertainty (H.2.)

· Generalizability and transferability of the economic evaluation results (I.2.)

· Ethical and distributional issues (I.3.)

In economic evaluations of newborn hearing screening, one of the main problems refers to the time horizon over which relevant costs and consequences accrue. In most of the studies, the time horizon from the initial screening test up to and including diagnostic follow-up was considered. The time horizon of seven economic evaluations was even restricted to the initial screening test [50,51,53,54,58,59,64]. To assess the cost-effectiveness of newborn hearing screening vs. risk or no (regular) screening, a long time horizon and a transsectoral perspective are needed, because long-term cost savings due to early detection and treatment are related to other sectors than the health care sector – such as, for example, education and labour. Four studies [24,25,60,63] used a societal perspective, three of which [24,25,60] considered a lifetime time horizon, but all long-term estimates heavily relied on assumptions rather than long-term empirical data. To date, the long-term effectiveness of newborn hearing screening programmes has not been adequately evaluated. In particular, valid data on patient-relevant parameters, such as social skills, language acquisition, educational development (e.g., school performance), professional career, and quality of life are sparse [66-69].

In general, the economic evaluations in newborn hearing screening lack a clear and detailed description of which cost categories were considered, how the associated resource quantities were measured, and how the resources were valued, which hinders the generalizability and transferability of economic evaluation results. Most of the studies only include the costs of screening and diagnostic follow-up. Costs for the implementation of the newborn hearing screening programme are only considered in some studies [48,56,63,65]. Furthermore, costs of screening vary due to different factors, such as different screening technologies (OAE or AABR), different settings (inpatient or outpatient), number of stages in the initial screening process (one-stage or two-stage), different target populations (all newborns or newborns with risk factors), or detection of hearing loss (unilateral or bilateral). In addition, measurement and valuation of resources vary, which also hinders the comparability of cost-effectiveness between studies. Furthermore, most studies rely on resource estimates identified through literature review or collected retrospectively alongside effectiveness data, and report only average costs per child screened or per child detected.

Instead of identifying the effectiveness of newborn hearing screening in terms of a health outcome variable, the standard approach to measuring effectiveness in all but three studies simply was to count the number of children screened or detected. Two further studies [23,61] used the number of child months detected to emphasize the importance of early detection and intervention. There is only one study [52] using a health outcome measure called “quality-weighted detected child months”. This QALY-like measure is based on the assumption that children with hearing losses detected earlier would experience an improved quality of life; however, empirical data in support of this assumption are missing. Finally, only eight studies reported incremental cost-effectiveness ratios [23-25,55,60,62,63,65] which are relevant to decision making in health care.

### Economic evaluations in newborn screening for inherited metabolic disorders including MCADD by MS/MS

In newborn screening of inherited metabolic disorders, the guidelines were applied to twelve economic evaluations [70-81], of which an overview can be found in Additional file 2. These studies were heterogeneous in terms of disease prevalence, frequency of adverse outcomes (e.g., morbidity and mortality), metabolic disorders considered under MS/MS screening, and cost categories considered. In these economic evaluations, one (MCADD) up to 21 inherited metabolic disorders were included. The prevalence of MCADD alone ranged from 1.19 to 9 per 100,000 newborns. Five studies were from the United States [73,75-77,80], two from Canada [71,78], and one each from Australia [81], Finland [70], France [79], the United Kingdom [74], and the Netherlands [72]. Ten of the twelve studies provided incremental cost-effectiveness ratios [70-73,75,77-81]. In the remaining two studies MS/MS screening was found to be cost saving [74,76]. The incremental cost-effectiveness ratios varied widely due to different epidemiological and clinical parameters, different outcome measures (life-years gained vs. QALYs gained), and differences in the number of conditions screened under MS/MS and in the cost categories considered in the economic evaluations (costs of screening and diagnostic follow-up, costs of immediate and ongoing treatment, costs of non-medical care, costs of productivity losses). For instance, only five studies considered start-up costs of MS/MS screening [70,76-79].

The results of the application of the guidelines revealed that most of the economic evaluations met the following items:

· Study question (B.1.)

· Intervention (B.2.)

· Target population (B.4.)

In contrast, the following items were inadequately met or not considered at all:

· Model validation (C.7.)

· Health outcomes measured in monetary units (D.3.) and non-health outcomes (D.5.)

· Method of valuing health benefits (D.2.b)

· Measurement (E.1.b. and E.2.b.) and valuation (E.1.c. and E.2.c.) of resource data

· Justification of discount rates (F.3.)

· Generalizability and transferability of the economic evaluation results (I.2.)

· Ethical and distributional issues (I.3.)

In economic evaluations of newborn screening for inherited metabolic disorders, one of the main problems relates to the lack of epidemiological data, such as the frequency of adverse outcomes with and without screening in terms of morbidity and mortality [12,13]. A further common weakness refers to the health outcomes used in the economic analysis. In half of the studies, life-years gained were used as a health outcome measure, but mortality does not attach great importance to the prevention of morbidity [42]. In contrast, the quality adjusted life year (QALY) incorporates both mortality and morbidity in a single outcome measure. However, the validity of QALY estimates in newborn screening is limited [82-84]. In newborn screening-related cost-utility analyses of MS/MS, it is common to take published utility weights from studies of adults with neurological impairments caused by other disorders than metabolic ones [42], although parent preferences for pediatric outcomes are also available [85]. The application of these parent preferences to published pediatric cost-utility analyses showed that more than a third of these analyses would change if such utilities were used [86]. In a recent review it was found that QALY weights for neurological impairments vary widely in pediatric economic evaluations [87], an issue which is also related to the economic evaluations considered here. To date, appropriate health state classification instruments that consider the dynamics of child development are lacking, health state classification instruments suitable for use in children aged 5 years and younger have not been established, and the role of proxies for measuring and valuing children’s health-related quality of life is not fully understood [82]. However, a child-friendly version of the EQ-5D is now available, namely the EQ-5D-Y [88], which proved to be feasible, reliable, and valid [89]. Nevertheless, further research is needed in terms of the development of methods that consider the health benefits of parents, and the consequences of integrating different forms of utility measurement in children and adults [82] in order to address potential harms arising from early detection or false-positive test results.

## Discussion

To date, no specific guidelines for assessing and improving the methodological quality of economic evaluations in newborn screening have been developed. Only one study was found that established “a generalised model of cost-effectiveness appraisal“ in the area of newborn screening for rare metabolic conditions using MS/MS, in order to identify the key variables that need to be considered to estimate costs and effects of MS/MS and alternatives [13]: these key variables comprise general items, such as prevalence of the conditions screened for with and without screening, or sensitivity and specificity of MS/MS and alternatives for the various conditions, screening costs, treatment costs, downstream health costs, societal costs, mortality, and morbidity. These newborn screening-specific guidelines were developed to highlight the need for greater standardization in the methodological quality of newborn screening-related economic evaluations and to ensure that future economic evaluations in newborn screening are conducted consistently and appropriately in order to enhance the comparability, generalizability and transferability of economic evaluation results in newborn screening. Furthermore, the high economic and human burden of diseases that can be detected early through newborn screening programmes gives such programmes a high priority in resource allocation, and underscores the need for specific guidelines. The application of the guidelines revealed methodological flaws regarding perspective, time horizon, discounting, use of assumptions rather than data, measuring and valuing health outcomes and resource data, modelling in terms of uncertainty and validation, transparency of reporting, generalizability, and ethical and distributional issues. Using the Pediatric Quality Appraisal Questionnaire [90], a quality appraisal of pediatric health economic evaluations published between 1980 and 1999 revealed that these economic evaluations were of good quality for “economic evaluation”, “comparators”, “target population”, “discounting” and “conclusions”, and only of poor quality for “perspective”, “incremental analysis”, and “conflict of interest” [91]. In this study, it was found that the quality domains of the specific guidelines developed here are fulfilled differently by the economic evaluations in the areas of newborn screening for inherited metabolic disorders including MCADD by MS/MS and newborn hearing screening. For example, in opposite to the economic evaluations in newborn hearing screening, all economic evaluations of newborn screening for inherited metabolic disorders including MCADD by MS/MS provide incremental cost-effectiveness ratios.

The guidelines proposed have several limitations. First, they can be applied to all economic evaluations in newborn screening. Therefore, issues specific to certain diseases detectable by newborn screening or technologies of newborn screening are not addressed in these guidelines. For instance, there is controversy in the literature whether newborn screening for all detectable disorders with MS/MS is cost-effective [92,93]. This question, from which methodological and ethical issues arise, is common for screening technologies that can be used to detect multiple conditions such as MS/MS or DNA-based screening. Cipriano et al. [78] suggest that variation in MS/MS sensitivity and specificity to detect other disorders, different treatment costs, and different potential benefits from early diagnosis and treatment may require the independent evaluation of each disease. Furthermore, researchers have still to elaborate a methodology which enables the economic evaluation of health technologies with a one-test, many-disorders approach – i.e., the simultaneous detection of a panel of disorders in a single individual. Second, the guidelines should be considered a starting point for assessing and improving the methodological quality of economic evaluations in newborn screening. As methodological issues advance, the guidelines should be regularly updated. Regarding issues of modelling and transferability, more detailed assessments are available [21,43,94,95]. Furthermore, the guidelines do not result in a quality score whose value, however, may be critically assessed [96], but in a structured abstract which is an alternative method of communicating the information to decision makers. In a study by [Thurston et al. 97] it was found that decision makers prefer a more detailed structured abstract in addition to a very short summary.

## Conclusions

These guidelines were provided in order to assess and improve the methodological quality of economic evaluations in newborn screening. The application to economic evaluations in the areas of newborn hearing screening, and newborn screening for inherited metabolic disorders including MCADD by MS/MS highlights important issues of economic evaluations in these two areas of newborn screening and underscores the need for their greater consideration in future newborn screening-related economic evaluations. The variation in methodological quality of the economic evaluations in newborn screening which is detected through application of the guidelines, and which limits the comparability, generalizability and transferability of economic evaluation results, reveals the necessity for specific guidelines regarding the methods for conducting sound economic evaluations in newborn screening.

## Competing interests

The authors declare that they have no competing interests.

## Authors’ contributions

AL conducted the searches and the review of economic evaluations, participated in study appraisal, and took the lead in writing the manuscript. JJ initiated the project, developed the guideline, provided methodological supervision throughout the study and contributed to writing the manuscript. RH participated in the development of the guideline and contributed to methodological discussions. All authors read and approved the final manuscript.

## Pre-publication history

The pre-publication history for this paper can be accessed here:

http://www.biomedcentral.com/1472-6963/12/300/prepub

## Supplementary Material

Additional file 1 **Overview of economic evaluations in newborn hearing screening.** Data extraction of selected economic evaluations of newborn hearing screening. Click here for file

Additional file 2 **Overview of economic evaluations in newborn screening for inherited metabolic disorders including MCADD by MS/MS.** Data extraction of selected economic evaluations of newborn screening for inherited metabolic disorders including MCADD by MS/MS. Click here for file
