# Supplementary material for: Specific guidelines for assessing and improving the methodological quality of economic evaluations of newborn screening
Source: BMC Health Serv Res. 2012 Sep 4;12:300. doi: 10.1186/1472-6963-12-300 (PMC3459803; doi:10.1186/1472-6963-12-300)
Supplement: Additional file 1 — Overview of economic evaluations in newborn hearing screening. Data extraction of selected economic evaluations of newborn hearing screening. [file 1472-6963-12-300-S1.doc]

### Additional file 1 – Overview of economic evaluations in newborn hearing screening

| **Screening strategy** | **Source, year, country** | **Target population** | **Screening interventions** | **Time horizon/perspective** | **Type of economic evaluation** | **Results in USPPP$2011*** |
| --- | --- | --- | --- | --- | --- | --- |
| UNHS | Böttcher et al. [48], 2009, Germany | All newborns | 1. TEOAE/AABR/two-tier/detection of uni- and bilateral losses/hospital 2. AABR/one-tier/detection of uni- and bilateral losses/hospital | From the initial screening test up to and including diagnostic evaluation/health care provider | CEA | Costs per child screened   1. 24.59 2. 29.91   Costs per child detected:   1. 11,413 2. 13,881 |
|  | Boshuizen et al. [49], 2001, Netherlands | All newborns not admitted to neonatal intensive care units | 1. OAE/two-tier/detection of uni- and bilateral losses/child health clinic (base case) 2. AABR/two-tier/detection of uni- and bilateral losses/child health clinic 3. OAE/three-tier/detection of uni- and bilateral losses/child health clinic 4. OAE/two-tier/detection of bilateral losses only/child health clinic 5. OAE/two-tier/detection of uni- and bilateral losses/home visits only 6. OAE/two-tier/detection of uni- and bilateral losses/home visit + child health clinic | From the initial screening test up to and including diagnostic evaluation/payer | CEA | Costs per child screened:   1. 38.07 2. 53.55 3. 32.97 4. 30.96 5. 43.17 6. 36.61   Costs per child detected:   1. 52,820 2. 74,131 3. 45,535 4. 42,803 5. 58,467 6. 49,906 |
|  | Dort et al. [50], 2000, Canada | All newborns | 1. AABR/one-tier/detection of uni- and bilateral losses/hospital 2. DPOAE/one-tier/detection of uni- and bilateral losses/hospital 3. TEOAE/one-tier/detection of uni- and bilateral losses/hospital | Screening only/health care provider | CCA | Costs per child screened:   1. 36.68 2. 18.51 3. 22.54 |
|  | Gorga et al. [51], 2001, USA | All newborns | 1. AABR/one-tier/detection of uni- and bilateral losses/hospital 2. OAE/one-tier/detection of uni- and bilateral losses/hospital 3. OAE/AABR/two-tier/detection of uni- and bilateral losses/hospital | Screening only/health care provider | CCA | Costs per child screened:   1. 38.41 2. 39.04 3. 28.22 |
|  | Grill et al. [52], 2006, UK | All newborns | 1. TEOAE/AABR/two-tier/detection of bilateral losses only/hospital-based screening 2. TEOAE/AABR/two-tier/detection of bilateral losses only/community-based screening | 120 months/health care system | CEA/CUA | Costs per child detected:   1. 59,940 2. 49,783   Cost per quality-weighted child months detected:   1. 570 2. 517 |
|  | Heinemann, Bohnert [53], 2000, Germany | All newborns | 1. TEOAE (Echoscreen)/one-tier/detection of uni- and bilateral losses/hospital 2. AABR (ALGO-Portable)/one-tier/detection of uni- and bilateral losses/hospital 3. AABR (Evoflash)/one-tier/detection of uni- and bilateral losses/hospital 4. TEOAE (Echoscreen)/AABR (Evoflash)/two-tier/detection of uni- and bilateral losses/hospital 5. TEOAE (Echoscreen)/AABR (ALGO-Portable)/two-tier/detection of uni- and bilateral losses/hospital | Screening only/health care provider | CCA | Costs per child screened:   1. 24.63 2. 77.15 3. 55.57 4. 27.14 5. 28.10 |
|  | Iley, Addis [54], 2000, UK | All newborns | 1. TEOAE/AABR/two-tier/detection of uni- and bilateral losses/hospital (in- or outpatient) 2. AABR/AABR/two-tier/detection of uni- and bilateral losses/hospital (in- or outpatient) | Screening only/health care provider | CCA | Costs per child screened:   1. 42.25 2. 37.68 |
|  | Kezirian et al. [55], 2001, USA | All newborns | 1. AABR/AABR/two-tier/detection of uni- and bilateral losses/hospital 2. AABR/one-tier/detection of uni- and bilateral losses/hospital 3. OAE/OAE/two-tier/detection of uni- and bilateral losses/hospital 4. OAE then AABR/one-tier/detection of uni- and bilateral losses/hospital | From the initial screening test up to and including diagnostic evaluation/health care provider | CEA | Costs per child screened:   1. 33.83 2. 41.58 3. 21.33 4. 33.35   Costs per child detected:   1. 13,401 2. 15,645 3. 8,447 4. 13,210 |
|  | Lemons et al. [56], 2002, USA | All newborns | 1. TEOAE/minimum one-tier/detection of uni- and bilateral losses/hospital 2. AABR/minimum one-tier/detection of uni- and bilateral losses/hospital | From the initial screening test up to and including diagnostic evaluation/health care provider | CCA | Costs per child screened:   1. 95.93 2. 75.75 |
|  | Lenarz et al. [65], 2007, Germany | All newborns | 1. TEOAE/two-tier/detection of uni- and bilateral losses/principally inpatient 2. TEOAE/two-tier/detection of uni- and bilateral losses/principally outpatient 3. TEOAE/two-tier/detection of uni- and bilateral losses/in- and outpatient | From the initial screening test up to and including diagnostic evaluation/health care provider | CEA | Costs per child screened:   1. 20.80 2. 43.53 3. 28.90   Costs per child detected:   1. 20,064 2. 42,201 3. 28,144 |
|  | Lin et al. [58], 2007, Taiwan | Healthy newborns | 1. TEOAE/one-tier/detection of uni- and bilateral losses/hospital 2. AABR/one-tier/detection of uni- and bilateral losses/hospital 3. TEOAE/AABR/two-tier/detection of uni- and bilateral losses/hospital | Screening only/health care provider | CCA | Costs per child screened:   1. 12.95 2. 9.44 3. 10.91 |
|  | Lin et al. [59], 2005, Taiwan | Healthy newborns | 1. TEOAE/one-tier/detection of uni- and bilateral losses/hospital 2. TEOAE/AABR/two-tier/detection of uni- and bilateral losses/hospital | Screening only/health care provider | CCA | Costs per child screened:   1. 14.01 2. 12.46 |
|  | Neumann et al. [64], 2004, Germany | All newborns | 1. AABR (MB11)/one-tier/detection of bilateral losses only/hospital 2. AABR (Evoflash)/one-tier/detection of uni- and bilateral losses/hospital | Screening only/health care provider | CEA | Costs per child screened:   1. 9.56 2. 15.11 (one ear) and 18.30 (both ears)   Costs per child detected:   1. 4,778 2. 7,556 (one ear) and 9,151 (both ears) |
|  | Schopflocher et al. [25], 2007, Canada | All newborns | 1. OAE/one-tier/detection of bilateral losses only/hospital 2. AABR/one-tier/detection of bilateral losses only/hospital 3. OAE/AABR/two-tier/detection of bilateral losses only/hospital | From the initial screening test up to and including diagnostic evaluation and lifetime in sensitivity analyses/society | CEA | Costs per child screened:   1. 26.41 2. 40.92 3. Not reported   Costs per child detected:   1. 4,496 2. 4,586 3. 4,515 |
|  | Uus et al. [63], 2006, UK | All newborns | 1. TEOAE/AABR/two-tier/detection of bilateral losses only/hospital- or community-based screening 2. Infant distraction test screening/one-tier/detection of bilateral losses only/community-based screening | 10 years/society | CEA | Costs per child detected:   1. 76,056 2. 257,573 |
|  | Vohr et al. [57], 2001, USA | Healthy newborns | 1. TEOAE/one-tier/detection of bilateral losses only/hospital 2. AABR/one-tier/detection of bilateral losses only/hospital 3. TEOAE/AABR/two-tier/detection of bilateral losses only/hospital | From the initial screening test up to and including diagnostic evaluation/health care provider | CEA | Costs per child screened:   1. 48.91 2. 55.94 3. 56.35   Costs per child detected:   1. 24,460 2. 27,969 3. 28,179 |
| UNHS vs. RS | Kemper, Downs [62], 2000, USA | UNHS: all newborns  RS: newborns with risk factors | 1. UNHS using TEOAE/AABR/two-tier/detection of bilateral losses only/hospital 2. RS using TEOAE/AABR/two-tier/detection of bilateral losses only/hospital | From the initial screening test up to and including diagnostic evaluation/health care system | CEA | Costs per child detected:   1. 19,862 2. 5,319 |
| UNHS vs. RS vs. NS | Hessel et al. [61], 2003, Germany | UNHS: all newborns  RS: all newborns with risk factors  NS: not systematically screened | 1. UNHS using TEOAE/two-tier/detection of uni- and bilateral losses/hospital 2. RS using TEOAE/two-tier/detection of uni- and bilateral losses/hospital 3. NS | 10 years/health care system | CEA | Costs per child screened:   1. 32.23 2. 16.15 3. 9.91   Costs per child detected:   1. 21,488 2. 10,772 3. 6,617   Costs per child months detected:   1. 231 2. 135 3. 98 |
|  | Keren et al. [60], 2002, USA | UNHS: all newborns  RS: newborns with risk factors | 1. UNHS using TEOAE/AABR/two-tier/detection of bilateral losses only/hospital 2. RS using AABR/AABR/two-tier/detection of bilateral losses only/hospital 3. NS | Lifetime/society | CEA | Costs per child detected:   1. 32,813 2. 15,486 3. 3,527   Costs per deaf child with normal language outcomes:   1. 2,712,885 2. 3,033,041 3. 3,397,048 |
|  | Merlin et al. [24], 2007, Australia | UNHS: all newborns  RS: only newborns with risk factors  NS: case finding | 1. UNHS using OAE/AABR or AABR/AABR (two-tier/detection of uni- and bilateral losses/hospital) 2. RS using AABR/one-tier/ detection of uni- and bilateral losses/hospital 3. NS | From the initial screening test up to and including diagnostic evaluation, and lifetime/health care system and society | CEA | Costs per child screened:   1. OAE/AABR: 37.40*/39.48** or AABR/AABR: 40.52*/54.03** 2. 253.51*/264.94** 3. 0   Costs per child detected:   1. OAE/AABR: 11,420*/12,175** or AABR/AABR: 14,593*/19,519** 2. 14,108*/14,874** 3. 28,336   *AABR using probe tips  **AABR using couplers  Net long-term savings based on a birth cohort of 250,000 infants:  1) vs. 2): 51,013,334  1) vs. 3): 98,701,969 |
|  | Schnell-Inderst et al. [23], 2006, Germany | UNHS: all hospital born newborns  RS: all children admitted to NICU  NS: no regular screening | 1. UNHS using TEOAE/AABR/two-tier/detection of uni- and bilateral losses/hospital 2. RS using TEOAE/AABR/two-tier/detection of uni- and bilateral losses/hospital 3. NS | From the initial screening test up to and including diagnostic evaluation/health care system | CEA | Costs per child screened:   1. 23.27 2. 6.26 3. 3.62   Costs per child detected:   1. 15,513 2. 4,173 3. 2,414   Costs per child months detected:   1. 133.12 2. 37.43 3. 22.66 |
| AABR: automated auditory brainstem response, CCA: cost-consequences analysis, CEA: cost-effectiveness analysis, DPOAE: distortion product otoacoustic emission, NICU: neonatal intensive care unit, NS: no screening, OAE: otoacoustic emission, RS: risk screening, TEOAE: transient-evoked otoacoustic emission, UK: United Kingdom, UNHS: universal newborn hearing screening, USA: United States of America  *Data from included economic studies were converted to US$ using the purchasing power parities of the Organization for Economic Co-operation and Development and standardized to the year 2011 according to the US medical care specific inflation rates given by the Bureau of Labor Statistics | | | | | | |
