# Supplementary material for: Specific guidelines for assessing and improving the methodological quality of economic evaluations of newborn screening
Source: BMC Health Serv Res. 2012 Sep 4;12:300. doi: 10.1186/1472-6963-12-300 (PMC3459803; doi:10.1186/1472-6963-12-300)
Supplement: Additional file 2 — Overview of economic evaluations in newborn screening for inherited metabolic disorders including MCADD by MS/MS. Data extraction of selected economic evaluations of newborn screening for inherited metabolic disorders including MCADD by MS/MS. [file 1472-6963-12-300-S2.doc]

### Additional file 2 – Overview of economic evaluations in newborn screening for inherited metabolic disorders including MCADD by MS/MS

| **Conditions under tandem mass spectrometry (MS/MS) screening** | **Source, year, country** | **Screening interventions** | **Time horizon/perspective** | **Type of economic evaluation** | **Incremental cost-effectiveness ratio (ICER) in USPPP$2011*** |
| --- | --- | --- | --- | --- | --- |
| MCADD | Hamers et al. [79], 2011, France | MS/MS screening vs. NS | Lifetime/health care system | CUA | US$22,819/QALY |
|  | Prosser et al. [80], 2010, USA | MS/MS screening vs. NS | Lifetime/society | CUA | US$26,326/QALY |
|  | Tran et al. [71], 2007, Canada | MS/MS screening vs. NS | 77 years/health care system | CUA | US$2,843/QALY |
|  | Van der Hilst et al. [72], 2007, Netherlands | MS/MS screening vs. NS | Lifetime/society | CEA | US$2,225/LYG |
|  | Venditti et al. [73], 2003, USA | MS/MS screening vs. NS | 20 and 70 years/society | CEA/CUA | US$16,866/LYG (20 years) and US$460/LYG (70 years)  US$8,587/QALY (20 years) and US$153/QALY (70 years) |
| Screening for MCADD, LCHADD, GA 1, and PKU using MS/MS; screening for CAH using an automatic immunoassay system | Autti-Rämö et al. [70], 2005, Finland | MS/MS screening vs. conventional screening for CH alone | 16 years, health care system | CUA | US$8,036/QALY – US$37,259/QALY |
| MCADD, PKU, CAH, CH, BIOT, MSUD, GA, and HCY | Carroll, Downs [76], 2006, USA | MS/MS screening vs. NS | Lifetime/society | CUA | Dominant |
| 8 fatty acid β-oxidation disorders, 6 organic acidemias, 3 urea cycle disorders, and 4 amino acidemias | Cipriano et al. [78], 2007, Canada | 1. MS/MS screening for MCADD alone vs. NS 2. 10 metabolic disorders including MCADD vs. conventional screening for PKU | Lifetime/society | CEA | US$68,686/LYG for MCADD alone, and US$71,503/LYG for MCADD and 9 other metabolic disorders |
| 7 fatty acid β-oxidation disorders, and 7 organic acidemias | Insinga et al. [77], 2002, USA | MS/MS screening vs. NS | Lifetime/society | CUA | US$64,187/QALY for MCADD alone and US$23,386/QALY for MCADD and the 13 other metabolic disorders |
| Amino acidurias, organic acidurias, urea cycle disorders, MCADD and other fatty acid oxidation defects | Norman et al. [81], 2009, Australia | MS/MS screening vs. NS | Lifetime/health care system | CEA | US$11,823/LYG |
| MCADD and PKU | Pandor et al. [74], 2006, UK | MS/MS screening vs. conventional screening for PKU alone | Lifetime/health care system | CEA | Dominant |
| MSUD, MCADD and other disorders of fatty acid oxidation, GA 1, MMA or PPA, urea cycle disorders, and HCY | Schoen et al. [75], 2002, USA | MS/MS screening vs. NS | Lifetime, HMO | CUA | US$9,301/QALY |
| BIOT: biotinidase deficiency, CAH: congenital adrenal hyperplasia, CH: congenital hypothyroidism, CEA: cost-effectiveness analysis, CUA: cost-utility analysis, GA 1: glutaric acidemia type 1, HCY: homocystinurea, HMO: health maintenance organization, LCHADD: long-chain hydroxyl acyl-CoA dehydrogenase deficiency, LYG: life years gained, MCADD: medium-chain acyl-CoA dehydrogenase deficiency, MMA: methylmalonic academia, MSUD: maple syrup urine disease, MS/MS: tandem mass spectrometry, NS: no screening, PKU: phenylketonuria, PPA: propionic academia, QALY: quality adjusted life year, UK: United Kingdom, USA: United States of America  *Data from included economic studies were converted to US$ using the purchasing power parities of the Organization for Economic Co-operation and Development and standardized to the year 2011 according to the US medical care specific inflation rates given by the Bureau of Labor Statistics | | | | | |
